# Cognitive and affective empathy predict young children's involvement in bullying one year later

**DOI:** 10.1002/jcv2.70127

**Published:** 2026-04-25

**Authors:** Katerina Romanova, Eleanor Leigh, Julia R. Badger, Richard P. Hastings, Suzy Clarkson, Matthew R. Broome, Judy Hutchings, Lucy Bowes

**Affiliations:** ^1^ Department of Experimental Psychology University of Oxford Oxford UK; ^2^ Department of Education University of Oxford Oxford UK; ^3^ School of Social Policy and Society University of Birmingham Birmingham UK; ^4^ School of Psychology and Sport Science Bangor University Bangor Wales UK; ^5^ Institute for Mental Health School of Psychology University of Birmingham Birmingham UK

**Keywords:** bullying, child, empathy, longitudinal studies, propensity score, risk factors

## Abstract

**Background:**

Bullying is a prevalent phenomenon that can have an array of negative impacts on both victims' and perpetrators' long‐term health and wellbeing. Despite the widespread assumption that empathy should be a key target for anti‐bullying interventions, research examining this relationship is surprisingly scarce, and the evidence base informing the implementation of empathy‐focused interventions remains in its infancy. Using data from the Stand Together trial, we examined how affective and cognitive empathy predict the status of victim, bully, and bully‐victim.

**Methods:**

We used a longitudinal trial with data collected in 2021 and 2022 from 4660 UK primary school children aged 6–11 years, including measures of empathy, victimisation, and involvement in bullying. We used propensity score matching and multinomial logistic regression to explore how children's self‐reported empathy towards victims of bullying at baseline predicted their role in bullying at 1‐year follow‐up.

**Results:**

Consistent with existing literature, we found that low affective empathy was a significant predictor of bullying perpetration at follow‐up (Odds Ratios (OR) = 0.60, 95% CI [0.45, 0.81], *p* < 0.001), but so was low cognitive empathy (OR = 0.73, 95% CI [0.56, 0.95], *p* < 0.001). We also found that both high affective (OR = 1.34, 95% CI [1.23, 1.47], *p* < 0.001) and cognitive (OR = 1.33, 95% CI [1.23, 1.44], *p* < 0.001) empathy predicted later victimisation.

**Conclusion:**

The findings identify high empathy as a new risk factor for peer victimisation and confirm the role of low empathy as a predictor of later bullying perpetration. We discuss how these findings can inform the strategic integration of empathy training to enhance the effectiveness of bullying prevention efforts.

## Key Points

**What's known?**

- Empathy is a significant component of anti‐bullying interventions, yet little is known about the nature of the relationship between the components of empathy and different bullying roles.

**What's new?**

- Higher affective and cognitive empathy predict later victimisation. However, higher affective and cognitive empathy also predicted decreased odds of later bullying perpetration.

**What's relevant?**

- Future studies should explore the mechanisms of high empathy as a risk factor for victimisation.
- Anti‐bullying interventions should carefully assess to whom they target empathy training efforts, as increasing empathy may not benefit all children.

## Graphical abstract

The study is a secondary data analysis of the UK Stand Together trial, investigating whether affective empathy and cognitive empathy at baseline predict involvement in bullying 1 year later. Using causal inference methods on data from over 4000 primary school children, we found that high empathy was associated with a decreased risk of becoming a bully but an increased risk of becoming victimised.

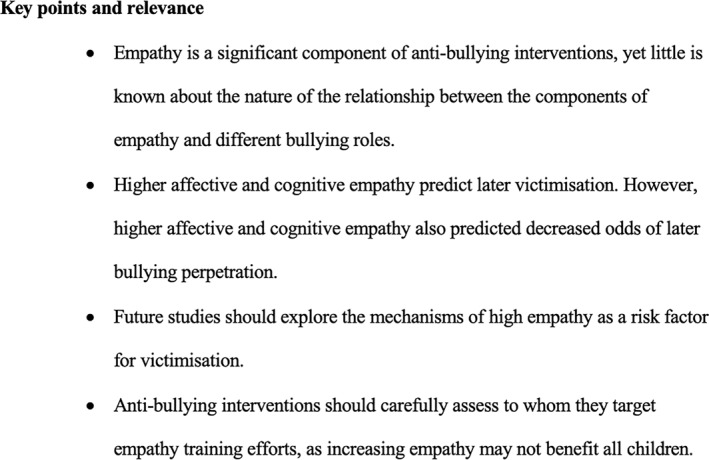

## INTRODUCTION

Childhood bullying is a major public health issue, commonly described as a deliberately harmful action repeated over time by an individual or a group who hold relatively more power (Sommerlad et al., 2021). Bullying can have profound and lasting consequences: victims of bullying are at a greater risk of mental health difficulties, in particular depression and anxiety (Arseneault et al., 2010), whilst perpetrators may struggle with externalising behaviour problems and a propensity for later substance abuse (Kaltiala‐Heino et al., 2000; Nansel et al., 2004). Bully‐victims, a term used to describe individuals who both perpetrate and experience bullying, experience the most adverse outcomes yet remain under‐researched (Marcos et al., 2024). Identifying risk and protective factors of bullying involvement is therefore crucial for prevention and early interventions.

Empathy is a common target in anti‐bullying interventions. Studies suggest that increasing empathy reduces antisocial behaviours such as bullying and promotes prosocial defending (Jolliffe & Farrington, 2004; Van Ryzin & Roseth, 2019). Empathy has two distinct dimensions; affective empathy, the ability to share another person's emotions (Mehrabian & Epstein, 1972), and cognitive empathy, the skill to recognise these emotions (Cohen & Strayer, 1996). These two dimensions may play distinct roles in peer victimisation (Fredrick et al., 2020). However, anti‐bullying interventions often consider empathy as a unitary construct in their implementation, and longitudinal research establishing how the two components relate to bullying roles in young children is scarce. It is essential to conduct studies on a younger population, as empathy develops substantially throughout childhood, as do the prevalence and type of bullying (e.g., Bowen & Holtom, 2010; Gaspar & Esteves, 2022).

The relationship between the components of empathy and bullying involvement is complex and context‐dependent (e.g., gender can affect this relationship as exemplified byAng & Goh (2010)). In cross‐sectional studies, lower levels of cognitive empathy have sometimes been associated with victimisation, as struggles with interpreting social cues might increase vulnerability (Balootbangan et al., 2023; van Noorden et al., 2015). However, cross‐sectional studies are limited in their ability to account for residual confounding and direction of effect and have traditionally not specified the target of empathy. Trach et al. (2023) conducted a longitudinal study of over 15,000 Finnish school children looking specifically at empathy towards the victim and their findings suggested that prolonged victimisation, in fact, increases cognitive empathy of victimised children, perhaps due to heightened identification with other victims. This new finding emphasises the importance of a longitudinal design, as well as considering the target of empathy, as empathy has been found to be target‐specific, rather than being a fixed trait (van Noorden et al., 2017). Affective empathy has been linked to victimisation, though its potential role in contributing to victimisation or moderating the relationship between victimisation and later psychopathology warrants further consideration (Malti et al., 2010).

Studies have consistently found the levels of affective empathy to be negatively associated with bullying perpetration in the general population, whereas findings on cognitive empathy are mixed (van Noorden et al., 2015). Some argue that higher cognitive empathy could enable bullies to exploit their victim's vulnerabilities (Sutton et al., 1999), whilst others suggest that low cognitive empathy could reduce the bullies' awareness of harm (Zych & Llorent, 2019). Bully‐victims have received comparatively little attention in research, despite the possibility that their empathy profiles may differ from those of pure bullies or victims. However, this has not yet been examined in longitudinal studies.

To address these gaps, our study leverages data from the ‘usual practise’ control arm of the Stand Together Trial (Bowes et al., 2024; Clarkson et al., 2022) that tested the effectiveness of the KiVa intervention, comprising a large longitudinal dataset investigating bullying in primary school children, collected at two time points over 1 year. Our study is unique in being the first longitudinal study on the topic, to use generalised propensity score matching, which allows us to adjust for multiple confounders and improve causal inferences. We matched based on age and gender at baseline, to control for their associations with both bullying and empathy (Sommerlad et al., 2021) as well as emotional symptoms since increased sensitivity and internalising symptoms related to high empathy could make children more vulnerable to victimisation or even more likely to report it (Gollwitzer et al., 2015; Konrad et al., 2024; Tone & Tully, 2014). Another confounder we considered for matching is bullying involvement at baseline, to help us establish directionality of effects. Using self‐reported cognitive and affective empathy towards the victim at baseline, we aim to evaluate how empathy independently predicts involvement in bullying at follow‐up as victims, bullies, and bully‐victims. Findings will clarify the distinct roles of empathy dimensions in bullying involvement and thusly inform anti‐bullying interventions to target empathy more effectively where indicated, and support preventative efforts.

## METHOD

### Participants

All participants were in the control arm of the Stand Together Trial, which recruited 118 state‐maintained primary schools across four sites in England and Wales. From the original sample of 11,111 children who took part in the trial, 4660 children who provided baseline data in 2021 and in the 2022 follow‐up and were in schools assigned to the usual practise arm (schools without the experimental intervention) were eligible for this study (2341 female, mean age = 8.77 years, SD = 0.92 years, range 6–11 years).

### Study procedure

This study used longitudinal data from two waves of the Stand Together Trial. The trial collected data from 118 primary schools across England and Wales (ages 7–11 years). The baseline data were collected in April‐June of the 2020/2021 academic year, and follow‐up data were collected after 12 months in a second wave (April–June of the 2021/2022 academic year). The schools where the study took place were all mainstream UK state‐maintained primary schools with Key Stage 2 classes. Data were collected from both children and staff who consented to participate. None of the schools were implementing an active recognised anti‐bullying intervention at baseline, beyond their standard anti‐bullying policy, and did not participate in the experimental anti‐bullying intervention at follow‐up, but were free to continue practise as usual, including various measures against bullying. Both children and staff filled out questionnaires on bullying, general wellbeing, health, and behaviour of the children. The trial details can be found in the UK Stand Together Trial Protocol (Clarkson et al., 2022).

This study looked at linked data from the two waves of data collection as outlined above. Children provided their data in the form of a questionnaire in a classroom setting under the instruction of teams of researchers from the Stand Together Trial. The children were fully debriefed at the beginning of each session about the meaning and contents of the questionnaire, their anonymity, and their right to withdraw their assent at any point. The questionnaires only contained the pupil identification number, and the linkage list was handled externally and destroyed following data entry.

### Measures

#### Empathy for the victim scale

The predictors included in this study were measures of the two components of empathy, assessed by the children's self‐report on the Empathy for the Victim Scale (EFTVS; Kärnä et al., 2011). The EFTVS is a seven‐item questionnaire that measures two subscales (cognitive and affective empathy), wherein children rated their empathy towards the victim on a four‐level Likert scale (*0 = never*, *4 = always*), including three items indicating cognitive empathy (e.g., “*I can understand how the bullied child must feel*”) and four items indicating affective empathy (e.g., “*When a child is bullied and feels sad*, *I also feel sad*”). We averaged the scores on the relevant items to provide a total score for each empathy component, with higher numbers indicating greater empathy. Internal consistency for the empathy scales was assessed using McDonald's Omega, to account for potential differences in factor loadings when using a short scale. The affective and the cognitive empathy scales demonstrated acceptable reliability (*ω* = 0.78 and *ω* = 0.77, respectively).

#### The Olweus bully/victim questionnaire

To classify participant role outcome at follow‐up as well as bullying involvement at baseline, we used the Olweus Bully/Victim Questionnaire (OBVQ), a widely used self‐report bullying involvement survey with 22 items, with a five‐level Likert scale (*0 = it hasn't happened in the past couple of months*, *4 = several times a week*). There are 11 items indicating victimisation and 11 items indicating bullying. For categorisation, we followed the criteria and method proposed by (Solberg & Olweus, 2003) using a value of three on the scale (i.e., ‘*2 or 3 times a month*’) as a minimum cut‐off point to denote bullying involvement in each question. Most studies have used the global items of the OBVQ, which uses a single question for classification of bullying and a single question for victimisation (e.g., ‘*How often have you been bullied at school in the past couple of months?*’). The sensitive measure of the OBVQ consists of multiple items (10 items for victimisation and 10 items for bullying) with concrete examples of bullying behaviours (e.g., ‘*I was made fun of*, *or teased in a hurtful way*’). The sensitive measure of the OBVQ is more accurate (Lee & Cornell, 2009), therefore we opted to conduct the primary analysis using the sensitive items and replicated the analysis on the global items. The analysis which used global items to categorise bullying involvement can be seen in Appendix S1 for interest, including descriptive statistics (Table S1), the results for affective empathy (Table S2) and cognitive empathy (Table S3). Within the sensitive measure of the OBVQ, for a participant to be classified as a victim or bully, they had to score higher than three on at least one item of the victimisation/bullying section. Participants who fulfiled the criteria for both bullying and victimisation were classified as bully‐victims only. In our dataset, we used dummy coding to represent bullying involvement as four categories (neither, victim, bully, bully‐victim) with ‘Neither’ as the baseline category, denoting no bullying involvement.

The sensitive measure of bullying involvement includes scenarios which cover all recognised main types of bullying. The sensitive measure of the OBVQ showed high reliability using Cronbach's alpha (the traditional measure of internal validity of the OBVQ), both on the victimisation scale (*α* = 0.88), and on the bullying scale (*α* = 0.80).

#### Teacher Strengths and Difficulties Questionnaire

We used the emotional symptoms subscale of the teacher‐reported Strengths and Difficulties Questionnaire (TSDQ), which measures children's psychosocial problems, to measure the emotional symptoms of children at baseline. The TSDQ is a validated and reliable measure which has been shown to be a good measure of emotional symptoms as well as a predictor of later life psychopathology (Stone et al., 2010). The emotional symptoms subscale of the TSDQ comprises five items, each with a three‐item Likert scale to assess whether statements about the child's emotional symptoms are true (*0 = Not true*, *1 = Somewhat true*, *2 = Certainly true*). We followed the proposed calculation of the questionnaire subscale by Goodman (1997), where scores on items which are indicative of emotional symptoms are added (e.g., “*Many worries*, *often seems worried*”). The TSDQ emotional subscale was used as a covariate to generate propensity scores. The internal consistency of the emotional symptoms subscale of the TSDQ was high (*ω* = 0.83) measured by McDonald's omega, which we used to account for differing factor loadings across items to provide a less biased reliability estimate for the short scale.

### Statistical analysis

All analyses were conducted in *R* (version 2023.09.1 + 494), using the tidyverse, mice, CausalGPS and nnet packages. Before analysis, we restricted the sample to children who had provided consent at both baseline and follow‐up (90.18% of the original sample) in the usual practise arm. To eliminate any missingness in the rest of the dataset, as is the prerequisite for propensity score matching, we used the MICE package in *R* (Van Buuren & Groothuis‐Oudshoorn, 2011) to perform multivariate imputation by chained equations and imputed the entire dataset using predictive mean matching to generate a total of 20 imputations with five iterations each, with the random seed set to 2025. Subsequent regression analyses were run on each imputed dataset separately and results were pooled following Rubin's rules.

We provided descriptive statistics reporting means and standard deviations to report the prevalence of different bullying roles, age, gender and levels of empathy. We used propensity score matching, and multinomial logistic regressions to test our hypotheses. We chose to use propensity score matching to simulate the characteristics and control of an experimental design in a natural sample and setting, where it is otherwise impossible to manipulate the independent/exposure variable. The predictor variables were the two separate continuous measures of cognitive empathy and affective empathy at baseline. The outcomes were the participant roles at follow‐up expressed categorically: Other (0), bully (1), victim (2), and bully‐victim (3). The covariates included to provide the basis for propensity score matching were age, gender, bullying involvement at baseline, and emotional symptoms.

We estimated propensity scores for each affective and cognitive empathy, based on our baseline covariates, generating generalised propensity scores (GPS), which are specifically designed for continuous predictor variables and are estimated using regression models. Wu et al. (2018) demonstrate the advantages of using GPS over simply including covariates in an outcome model, particularly because they increase objectivity by separating design from analysis, and provide better opportunities to assess covariate balance. We used the CausalGPS package in *R* (Khoshnevis et al., 2023) to generate GPS using a continuous exposure to approximate a causal design with an observational dataset (Wu et al., 2018). Next, we checked for the balance of covariates across the GPS by calculating absolute correlation and block absolute standardised bias for continuous exposures, as suggested by Wu et al. (2018). Given that our covariates were well‐balanced (see Table S4 within Appendix S2), we proceeded to the analysis stage.

To conduct the causal inference analysis, we ran multiple multinomial logistic regressions to examine our research question, using the GPS as a covariate. The predictor variables were the two continuous measures of empathy (cognitive and affective) at baseline. The outcomes were the participant roles at follow‐up expressed categorically: Other (0), victim (1), bully (2), and bully‐victim (3). We ran a directional null hypothesis significance test to see whether cumulative empathy at baseline is associated with later involvement in bullying over and above the comparator group (which consists of those involved neither as bullies, victims, nor bully‐victims). Regression was run individually for each imputed dataset and model estimates were then pooled across imputations and reported as Odds Ratios (OR) with a 95% confidence intervals.

To check whether clustering by schools influenced our results, we ran an exploratory analysis to test its effect. We ran a Bayesian mixed‐effect multinomial logistic regression with a random intercept for the “school ID” variable for each imputed dataset and pooled our results, which did not change our findings and are reported in Appendix S3 for transparency. The results for affective empathy are reported in Table S5 and the results for cognitive empathy are reported in Table S6 within Appendix S3.

## RESULTS

### Descriptive statistics

The descriptive statistics regarding bullying involvement according to the specific measure are summarised in Table 1. Most children were not involved in bullying (53.13%). Some statistics of note include the higher prevalence for males in the roles of bully (54.81%) as well as bully‐victim (60.42%). Interestingly, cognitive empathy at baseline was slightly lower for bullies (*M* = 1.72, SD = 0.82) than non‐involved children (*M* = 1.75, SD = 0.82), whilst both victims and bully‐victims at baseline showed higher levels of cognitive empathy than non‐involved children (*M* = 1.95, SD = 0.81; *M* = 1.83, SD = 0.84). Affective empathy was also slightly lower for bullies at baseline (*M* = 1.46, SD = 0.80), but higher for victims at baseline (*M* = 1.67, SD = 0.75) than children not involved in bullying (*M* = 1.49, SD = 0.74), who showed lower levels of affective empathy than bully‐victims (*M* = 1.54, SD = 0.81).

**TABLE 1:** Descriptive statistics based on the sensitive classification.

Role	Prevalence (*N*)	Male (%)	Female (%)	Mean age	SD age	Mean cognitive empathy	SD cognitive empathy	Mean affective empathy	SD affective empathy
Neither	2476	49.47	50.53	8.82	0.92	1.75	0.82	1.49	0.74
Bully	270	54.81	45.19	8.64	0.96	1.72	0.82	1.46	0.80
Victim	1583	47.82	52.18	8.74	0.92	1.95	0.81	1.67	0.75
Bully‐victim	331	60.42	39.58	8.66	0.92	1.83	0.84	1.54	0.81

*Note*: Prevalence, gender percentages, mean age, and empathy scores (mean and SD) are provided for each role. Role categories indicating bullying involvement include Neither (not involved), Bully, Victim, and Bully‐Victim.

### Empathy and bullying involvement

The regression model, the outcomes of which are in Table 2, showed that levels of cognitive empathy were significantly associated with both bullying and victimisation. Children with higher cognitive empathy were significantly more likely to be victimised 1 year later when GPS were considered (OR = 1.33, 95% CI [1.23, 1.44], *p* < 0.001). Specifically, a one‐unit increase in the average affective empathy score would mean that the multinomial log‐odds of being a victim are 1.33 units higher. There was no relationship between empathy and later status of a bully‐victim. Finally, a higher score of cognitive empathy was associated with reduced odds of becoming a bully 1 year later (OR = 0.73, 95% CI [0.56, 0.95], *p* < 0.05).

**TABLE 2:** Affective empathy predicting bullying involvement according to the sensitive measure of bullying.

Outcome	*b* (log odds)	SE	*p*	OR	95% CI
Bully					
Affective empathy	−0.393	0.148	< 0.05	0.607	[0.453, 0.811]
Victim					
Affective empathy	0.341	0.045	< 0.001	1.341	[1.227, 1.465]
Bully‐victim					
Affective empathy	0.072	0.076	0.361	1.072	[0.924, 1.243]

*Note*: Reference level is not involved. Odds Ratios (OR) are exponentiated coefficients.

Affective empathy at baseline significantly predicted later victimisation when GPS were included as covariate (OR = 1.34, 95% CI [1.23, 1.47], *p* < 0.001). A one‐unit increase in total affective empathy score was associated with a 1.34 times higher likelihood of being a victim than not being involved in bullying. High affective empathy also significantly reduced the odds of later becoming a bully (OR = 0.61, 95% CI [0.45, 0.81], *p* < .05). A one‐unit increase in the affective empathy score was associated with a 49% decrease in the likelihood of becoming a bully. There was no significant relationship between affective empathy and involvement as a bully‐victim (see Table 3 for results).

**TABLE 3:** Cognitive empathy predicting bullying involvement according to the sensitive measure of bullying.

Outcome	*b* (log odds)	SE	*p*	OR	95% CI
Bully					
Cognitive empathy	−0.271	0.137	< 0.05	0.729	[0.557, 0.953]
Victim					
Cognitive empathy	0.330	0.040	< 0.001	1.330	[1.230, 1.439]
Bully‐victim					
Cognitive empathy	0.106	0.072	0.162	1.106	[0.960, 1.275]

*Note*: Reference level is not involved. Odds Ratios (OR) are exponentiated coefficients.

## DISCUSSION

We aimed to investigate whether affective and cognitive empathy towards a victim independently predict later involvement of children in bullying in primary schools, whilst adjusting for important confounders. We used a longitudinal dataset and GPS to investigate whether there was a causal relationship between levels of empathy and becoming a victim of peer bullying, a bully, or a bully‐victim. We found that children with higher levels of cognitive and affective empathy were more likely to experience later victimisation than children with lower levels of empathy. Furthermore, the results showed that having low cognitive and affective empathy might contribute to a child becoming a bully. Neither cognitive nor affective empathy predicted children's likelihood of being a bully‐victim.

One of the most robust findings of the study suggests that highly empathetic individuals (both in terms of cognitive and affective empathy) are at increased risk of later victimisation. The relationship between high empathy and victimisation has previously been found only in cross‐sectional studies, and primarily in studies of cyber‐bullying (DeSmet et al., 2016; Zych & Llorent, 2019). Previously, it has been argued that this is a result of continuous exposure to victimisation (Fabris et al., 2022), which has even been found in longitudinal studies (Trach et al., 2023). However, our study adjusted for bullying at baseline and found that when multiple other factors, including emotional symptoms, are accounted for, high empathy is shown as a risk factor for becoming victimised. The discrepancies in findings could be explained due to a higher number of confounders considered in this study, or the fact that the sample consisted only of primary school children. The finding warrants further exploration, as it poses a potential challenge to the widespread use of non‐targeted empathy training in schools as a means of anti‐bullying measures. Furthermore, recent research has suggested that affective empathy may be associated with negative outcomes, specifically related to mental health problems, which reinforces the idea that a “one size fits all” approach could pose problems (Yan et al., 2021).

We also confirmed the common finding that low affective empathy is associated with an increased likelihood of perpetrating bullying. This is consistent with findings of past meta‐analyses (e.g., van Noorden et al., 2015), adding strength to claims already made by cross‐sectional studies. The finding is also consistent with the theory which drives the inclusion of empathy training as a component of anti‐bullying interventions, which is that low affective empathy drives antisocial behaviour, making a bully less likely to experience an adverse reaction to their victim's distress. Furthermore, our results contribute to the debate on the role of cognitive empathy in bullying, which we found to negatively predict later bullying. This is in line with the proposal that bullies might not be fully aware of the impact of their actions on the victim, making them more likely to engage in bullying without consequent guilt. These findings would encourage the continuation of efforts to increase affective and cognitive empathy towards victims of bullying as a preventative measure against future bullying. Of note is also the surprising finding that we did not see an association between empathy at baseline and likelihood of becoming a bully‐victim. It is unusual to find a weaker association with risk factors for bullying‐victimisation compared to other forms of direct involvement, but since this is the first study to consider this relationship, more research is needed to explore this question. A considerable strength of our study is the use of generalised propensity score matching with a longitudinal design, enabling some causal inference, and including multiple covariates to account for confounding. However, it is important to note that our matches were limited by the kind of data collected in our study. Of interest is the fact that our study is one of the first to consider a large and diverse primary school sample, elucidating the relationship between empathy and bullying at an earlier age. This is important not only because bullying is already prevalent at this age, but also because empathy is still developing and might therefore be particularly malleable. Furthermore, the study confirms the importance of the argument made by Trach et al. (2023) to include empathy towards the victim specifically, as this is most relevant to the bullying scenario and might explain differences in results as empathy is target specific.

Some limitations of our study concern the measures used. First of all, our study relied on self‐reported bullying, which is not always reliable, particularly as a result of social desirability bias. Future studies could benefit from using a range of bullying measures such as peer‐reported bullying to complement the measure. Furthermore, measuring empathy through self‐reported questionnaires at a young age also poses challenges. Children might struggle to understand questions requiring mentalising at an age where this ability is still developing. Furthermore, it might be difficult for a person to assess their ability to identify other's emotions credibly. Further research would benefit from exploring options for measuring empathy in such a young sample, such as using demonstrative scenarios using qualitative tools to increase understanding (Garton & Gringart, 2005). The next steps will also include testing the mechanisms by which empathy might be a risk factor to later victimisation and exploring how the previously reported association between empathy and internalising symptoms could add to this risk (Tone & Tully, 2014).

We hope our study will inform interventions against bullying and add to our understanding of what factors might contribute to bullying and victimisation. Current interventions are training empathy, largely on an intuitive belief that more empathy is always good, but this does not always consider those directly involved in bullying, nor the complex nature of empathy. Our findings suggest that a targeted approach to training relevant components of empathy might be more helpful. The identification of empathy as a potential risk factor for bullying involvement warrants further investigation, with more studies focussing on primary school children.

## AUTHOR CONTRIBUTIONS

**Katerina Romanova**: Conceptualization; data curation; formal analysis; investigation; methodology; visualization; writing—original draft; writing—review and editing. **Eleanor Leigh**: Conceptualization; methodology; supervision; writing—review and editing. **Julia R. Badger**: Conceptualization; funding acquisition; investigation; writing—review and editing. **Richard P. Hastings**: Conceptualization; investigation; writing—review and editing. **Suzy Clarkson**: Conceptualization; writing—review and editing. **Matthew R. Broome**: Conceptualization; investigation; writing—review and editing. **Judy M. Hutchings**: Conceptualization; writing—review and editing; **Lucy Bowes**: Conceptualization; funding acquisition; investigation; methodology; project administration; supervision; writing—review and editing.

## CONFLICT OF INTEREST STATEMENT

The authors declare no conflicts of interest.

## ETHICAL CONSIDERATIONS

Ethical approval for the Stand Together Trial was granted by Bangor University Psychology Reseearch Ethics and Governance Committee (reference: 2019–16592) on 13th November 2019. Ethical approval for the specific study was granted by the Medical Sciences Interdivisional Research Ethics Committee (MS IDREC) of the University of Oxford (reference: R83987/RE001) on 17th November 2022. All head headteachers gave written consent for the participation of their schools in the trial. Teaching staff provided written consent for their participation. Parents of pupils were informed by email about the trial and given the opportunity to opt‐out their children via electronic opt‐out. Children provided electronic informed consent at each data collection point.

## Supporting information

Supporting Information S1

## Data Availability

De‐identified participant data from this study along with a data dictionary can be made available after a signed data access agreement upon reasonable request to the principal investigators of the trial (j.hutchings@bangor.ac.uk and lucy.bowes@psy.ox.ac.uk).
