# Supplementary material for: Cognitive and affective empathy predict young children's involvement in bullying one year later
Source: JCPP Adv. 2026 Apr 25:e70127. Online ahead of print. doi: 10.1002/jcv2.70127 (PMC13339301; doi:10.1002/jcv2.70127)
Supplement: Supplementary file 1 — Supporting Information S1 [file JCV2-9999-e70127-s001.docx]

**Cognitive and Affective Empathy Predict Young Children’s Involvement in Bullying One Year Later**

**Supporting Information**

**Appendix S1. Global Measure of Bullying**

The descriptive statistics regarding bullying involvement according to the global measure are summarised in *Table S1*. According to this measure, most children were still not involved in bullying, with numbers going down across all forms of bullying involvement as opposed to the specific measure. We could once again see the slightly higher prevalence of males in the roles of bully (55.87%) as and bully-victim (60.34%). Victims (M = 8.63 years, SD = 0.92) and bullies (M = 8.63 years, SD = 0.97), were on average slightly younger than those classified as bully-victims (M = 8.83 years, SD = 0.86). Cognitive empathy at baseline was lower for bullies (M = 1.76, SD = 0.92) than non-involved children (M = 1.78, SD = 0.81), who showed lower levels of cognitive empathy than bully-victims (M = 1.80, SD = 0.80), whilst victims showed higher levels of cognitive empathy than any other groups at baseline (M = 2.02, SD = 0.82). Affective empathy was once again lower for bullies at baseline (M = 1.49, SD = 0.85), similar to bully-victims (M = 1.36, SD = 0.79) and higher for victims at baseline (M = 1.75, SD = 0.77) than for children not involved in bullying (M = 1.59, SD = 0.74).

**Empathy and Bullying Involvement Using the Global Measure**

The multinomial logistic regression showed that high cognitive empathy significantly predicted later victimisation (*OR* = 1.39, 95% CI [1.26, 1.53], *p* < .001), which can be seen in *Table S2*, as did high affective empathy (*OR* = 1.45, 95% CI [1.30, 1.61], *p* < .001), as represented in *Table S3*. A one-unit increase in total cognitive empathy score was associated with a 1.39 times higher likelihood of being a victim compared to the comparator group, and 1.45 as likely with a one-unit increase in the total score of affective empathy. There were no significant relationships between either type of empathy and involvement as bully or bully-victim.

**Appendix S2. Covariate Balance**

Below in Table S4 we report the Block-Average Absolute Standardized Bias (BASB) table to demonstrate balance of the covariates following propensity matching. The values demonstrate acceptable balance across all covariates for the generalised propensity scores for both measures of empathy.

**Appendix S3. Clustering Analysis**

Below we report the results of a Bayesian mixed-effect multinomial logistic regression which included random intercepts for the “School ID” variable, in order account for the nested nature of data and any potential clustering by schools whilst testing our research question about the influence of cognitive and affective empathy on later bullying involvement. This exploratory analysis confirms that our earlier findings hold true, even when clustering by schools is controlled for. Results for affective empathy are reported in Table S5 and results for cognitive empathy are reported in Table S6.
